# The effects of weight gain after smoking cessation on atherogenic α1-antitrypsin–low-density lipoprotein

**DOI:** 10.1007/s00380-014-0549-9

**Published:** 2014-08-03

**Authors:** Maki Komiyama, Hiromichi Wada, Shuichi Ura, Hajime Yamakage, Noriko Satoh-Asahara, Sayaka Shimada, Masaharu Akao, Hiroshi Koyama, Koichi Kono, Akira Shimatsu, Yuko Takahashi, Koji Hasegawa

**Affiliations:** 1 Department of General Internal Medicine, National Hospital Organization Kyoto Medical Center, Kyoto, Japan; 2 Division of Translational Research, National Hospital Organization Kyoto Medical Center, 1-1 Mukaihata-cho, Fukakusa, Fushimi-ku, Kyoto, 612-8555 Japan; 3 Clinical Research Institute, National Hospital Organization Kyoto Medical Center, Kyoto, Japan; 4 Department of Cardiology, National Hospital Organization Kyoto Medical Center, Kyoto, Japan; 5 Department of Hygiene and Public Health, Osaka Medical College, Osaka, Japan; 6 Health Care Center, Nara Women’s University, Nara, Japan

**Keywords:** Smoking cessation, Obesity, Oxiditive stresss, Atherosclerosis, Prevention

## Abstract

Although cardiovascular risks decrease after quitting smoking, body weight often increases in the early period after smoking cessation. We have previously reported that the serum level of the α1-antitrypsin–low-density lipoprotein complex (AT–LDL)—an oxidatively modified low-density lipoprotein that accelerates atherosclerosis—is high in current smokers, and that the level rapidly decreases after smoking cessation. However, the effects of weight gain after smoking cessation on this cardiovascular marker are unknown. In 183 outpatients (134 males, 49 females) who had successfully quit smoking, serum AT–LDL levels were measured using an enzyme-linked immunosorbent assay. For all persons who had successfully quit smoking, body mass index (BMI) significantly increased 12 weeks after the first examination (*p* < 0.01). Among patients with a BMI increase smaller than the median, a significant decrease (*p* < 0.01) in serum AT–LDL values was found, but no significant changes in serum AT–LDL values were found in patients with a BMI increase greater than the median. The findings suggest that the decrease in serum AT–LDL levels after quitting smoking is influenced by weight gain after smoking cessation.

## Introduction

Smoking and obesity are both independent risk factors for cardiovascular diseases; thus smokers are encouraged to quit smoking and obese persons to decrease their weight. Although the cardiovascular risks begin to decline within 2 years after quitting smoking, it takes 10 years or longer for the risk levels to decline to those of nonsmokers [1]. Moreover, weight gain is commonly noted for at least a few years after smoking cessation [2], and obesity incidence among many patients, including those with metabolic syndrome, may increase when smoking cessation is attempted [3, 4]. The reported extent of weight gain among individuals who quit smoking varies, but on average, males gain 2.8 kg and females gain 3.8 kg, with over 10 % of such persons experiencing weight gain of ≥13 kg [3]. Recently, it was reported that as long as weight gain after smoking cessation is kept under 5 kg, the risk of cardiovascular events does not increase compared with smokers [5]. However, as the risk reduction benefit is small in the early period of smoking cessation, there is a possibility that body weight gain in this period temporarily increases the risk of cardiovascular disease. Nevertheless, not enough is known regarding the effects of weight gain in the early period after smoking cessation on cardiovascular risks.

Smoking causes increased oxidative stress and induces lipid oxidation [6–8]; oxidized low-density lipoproteins (LDLs), in turn, contribute to the initiation and progression of atherosclerotic lesions [9, 10]. One novel oxidized LDL is α1-antitrypsin–low-density lipoprotein (AT–LDL), which is a complex of oxidized α1-antitrypsin (AT) and LDL [11]. AT is a serine proteinase inhibitor that protects tissues by inhibiting proteinase; however, when AT is oxidized, it loses its tissue-protective action [12]. Oxidized AT also recruits and activates monocytes [12]. The AT–LDL complex can be found in human serum and at atherosclerotic lesion sites [13]; serum AT–LDL levels are thought to reflect foam cell activity within atherosclerotic lesions [13].

We have previously reported that AT–LDL serum levels are significantly higher in current smokers than in both nonsmokers and former smokers; that the increase in AT–LDL is proportional to smoking duration; and that AT–LDL levels rapidly decrease after smoking cessation [14]. These facts suggest that AT–LDL has a close relationship with smoking and that it may be a useful indicator of oxidative stress in smokers. Both smoking and obesity have a close relationship with oxidative stress, and both are linked with cardiovascular diseases. However, the contributions of smoking cessation and obesity to oxidative stress during post-smoking cessation weight gain are unknown. In addition, their influences on markers of cardiovascular disease and oxidative stress have not been clarified. To investigate the effects of weight gain in the early period after smoking cessation on AT–LDL levels, we measured body mass index (BMI) and AT–LDL at baseline and 12 weeks after beginning a smoking cessation program.

## Materials and methods

### Subjects

This is a retrospective study, in which we gathered data on patients who visited the Smoking Cessation Clinic at the National Hospital Organization Kyoto Medical Center between April 2007 and June 2013. Among the 201 patients who successfully quit smoking, 18 patients were excluded because of the lack of body weight data. Therefore, we analyzed the findings of 183 patients (134 males and 49 females, aged between 22 and 81 years, 61 ± 12 years on average). During the smoking cessation program, 46 patients received antihypertensive agents, 27 received statins, and 20 received medications for diabetes mellitus. Various parameters were evaluated in these patients at the time of the initial consultation and after smoking cessation (12 weeks after the initial consultation).

The objective of the study was explained to the subjects in writing, and all participants provided written informed consent. The protocol was approved by the Ethical Review Board, National Hospital Organization Kyoto Medical Center.

### Smoking cessation clinic

At the initial consultation, nicotine dependence was assessed with the Fagerström test for nicotine dependence (FTND) [15]. Scores range from 0 to 10, with higher scores indicating more severe nicotine dependence. The number of cigarettes smoked per day was determined by asking the smoker, “On average, in the past month, how many cigarettes did you smoke per day?” Smokers were asked to rate their confidence in their ability to abstain from smoking cigarettes over the next 12 weeks on a scale from 0 to 100 %.

Anti-smoking treatment was conducted according to the standard procedures for anti-smoking treatment, originally issued in March 2006 by nine academic societies including the Japanese Circulation Society, Japan Lung Cancer Society, and Japanese Cancer Association. The patients were examined at their first visit and 2, 4, 8, and 12 weeks thereafter, and they were treated with either nicotine patches or oral varenicline. At each visit maintenance of smoking cessation was ascertained, and specific advice concerning the continuation of cessation was given. At the end of the 12-week anti-smoking treatment, smoking cessation maintenance was evaluated again. A patient was judged to have succeeded in quitting smoking with an expiratory carbon monoxide (CO) concentration of 7 ppm or less and the patient’s verbal affirmation of no smoking. Expiratory CO concentration was measured using the EC50 Micro Smokerlyzer^R^ (Bedfront Scientific Ltd., Kent, UK), which measures the end-tidal CO electrochemically, with a reported accuracy of ±2 % [16]. The attempt to quit smoking was judged to have been unsuccessful if the patient discontinued treatment or continued visiting but failed to quit smoking.

### Data collection

A patient’s BMI was calculated as the weight in kilograms divided by the square of the height in meters. Patients were instructed to weight themselves at the same time each day. Systolic and diastolic blood pressures were measured with an automatic electronic sphygmomanometer (BP-103iII; Nippon Colin, Komaki, Japan) [17] and the regular-sized cuff for Japanese patients (appropriate arm length 17–32 cm) as recommended.

### Blood sampling

Blood was obtained from each patient’s antecubital vein 2–3 h after lunch to measure the plasma levels of hemoglobin A_1c_ (HbA_1c_) and serum levels of low-density lipoprotein cholesterol (LDL-C), high-density lipoprotein cholesterol (HDL-C), and triglycerides (TG). Plasma levels of HbA_1c_ and serum levels of HDL-C and LDL-C were measured using an automatic analyzer (LABOSPECT 008; Hitachi High Technologies Co., Ltd., Tokyo, Japan) with enzyme-based reagents (Kyowa Medex Co., Ltd., Tokyo, Japan). Serum levels of AT–LDL and serum amyloid A-LDL (SAA-LDL) were measured employing specific sandwich enzyme-linked immunosorbent assays (Ikagaku Co., Ltd., Kyoto, Japan). Mixed solutions of very-low-density lipoprotein and LDL isolated from serum by polyanion precipitation [18] were measured using anti-human polyclonal antibodies against SAA or AT (DAKO Denmark A/S, Glostrup, Denmark) as the primary antibodies and anti-human apoB monoclonal antibody (SAA-LDL, clone No. 427; AT–LDL, clone No. 27) as the secondary antibody. The intra-assay coefficients of variation at low and high levels of SAA-LDL were 2.6 and 4.7 %, respectively. The intra-assay coefficients of variation at low and high levels of AT–LDL were 1.8 and 1.6 %, respectively. The interassay coefficients of variation at low and high levels of SAA-LDL were 5.0 and 6.7 %, respectively. The interassay coefficients of variation at low and high levels of AT–LDL were 5.9 and 5.4 %, respectively. The intra-assay and interassay coefficients of variation of SAA were 7.4 and 7.8 %, respectively. The intra-assay and interassay coefficients of variation of AT were 4.1 and 7.0 %, respectively. These assays were performed by an investigator blinded to the sources of the samples.

### Evaluation of depression

The severity of depression was evaluated using a questionnaire based on the self-rating depression scale (SDS). The questionnaire was answered by the patients themselves, the answers were checked by the study staff, and the patients were asked to fill it in again if there were items that they had omitted or mistakes had been made on answering [19, 20].

### Statistical analysis

All statistical analyses were performed using SPSS Statistics 17.0 statistical software package (SPSS Inc., Chicago, IL, USA). Data are presented as the mean ± standard deviation (SD), and *p* < 0.05 was considered significant. Statistical analyses were performed as previously described [21]. Clinical data before and after successful smoking cessation were compared with the paired *t* test or the Wilcoxon signed rank test. In addition, changes in data from before to after smoking cessation were compared by two-way analysis of variance (ANOVA) between patients with a BMI increase smaller than the median and those with a BMI increase greater than the median.

## Results

Table 1 compares data collected at the time of the first examination and after smoking cessation (12 weeks after the first examination). Compared with their baseline values, after smoking cessation patients experienced an increase in BMI (*p* < 0.001), LDL-C (*p* = 0.042), HDL-C (*p* < 0.001), and TG (*p* < 0.001), whereas they had a decrease in systolic blood pressure (SBP) (*p* = 0.012), AT–LDL (*p* < 0.001), and the CO concentration in exhaled breath (*p* < 0.001).

**Table 1:** Patient data before and after successful smoking cessation (*n* = 183)

	Before	After	*p* value
BMI (kg/m^2^)	23.4 ± 3.6	23.8 ± 3.8	<0.001 a
SBP (mmHg)	131 ± 19	127 ± 19	0.012 a
DBP (mmHg)	75 ± 11	73 ± 11	0.083 a
HbA_1c_ (%)	6.1 ± 1.0	6.2 ± 1.1	0.155 b
LDL-C (mg/dL)	113 ± 29	117 ± 30	0.042 a
HDL-C (mg/dL)	57 ± 17	60 ± 18	<0.001 a
TG (mg/dL)	169 ± 101	203 ± 129	<0.001 b
SAA-LDL (μg/mL)	19.0 ± 21.9	21.8 ± 37.8	0.350 b
AT–LDL (μg/mL)	2.7 ± 1.3	2.3 ± 0.9	<0.001 b
CO (ppm)	17.6 ± 14.7	1.8 ± 1.5	<0.001 b
SDS test score	38.4 ± 10.9	37.5 ± 10.9	0.195 b

Data are presented as the mean ± standard deviation

*BMI* body mass index, *SBP* systolic blood pressures, *DBP* diastolic blood pressures, *HbA*_*1c*_ hemoglobin A_1c_, *LDL-C* low-density lipoprotein cholesterol, *HDL-C* high-density lipoprotein cholesterol, *TG* triglycerides, *SAA-LDL* serum amyloid A-LDL, *AT–LDL* α1-antitrypsin–low-density lipoprotein, *CO* carbon monoxide, *SDS* test score, self-rating depression scale test score

*p* value: a, paired *t* test; b, Wilcoxon signed rank test

The median BMI increase rate from baseline to 12 weeks after starting smoking cessation therapy was 1.25 %. Using the median BMI increase as the cutoff point, participants were divided into two groups: those whose rate of change in BMI was below the median (ΔBMI < median) and those whose rate of change was at or above the median (ΔBMI ≥ median). Table 2 presents baseline data stratified by the ΔBMI group; no significant differences were noted between the groups at the time of the initial examination. Table 3 presents the results of a comparison of data before and after smoking cessation by the ΔBMI group. In the ΔBMI < median group, patients’ BMI decreased after smoking cessation by an average of 0.4 kg/m^2^ (*p* < 0.01), whereas in the ΔBMI ≥ median group, the BMI increased after smoking cessation by 1.1 kg/m^2^ (*p* < 0.01). There was no significant change in serum LDL-C levels over 12 weeks in the ΔBMI < median group, whereas a significant increase (*p* = 0.001) was found in the ΔBMI ≥ median group after smoking cessation. Conversely, although no significant change in the serum levels of AT–LDL was found in the ΔBMI ≥ median group, a significant decrease in AT–LDL levels (*p* < 0.001) from before to after smoking cessation was identified in the ΔBMI < median group. Moreover, the degree of change in serum AT–LDL levels was significantly larger (*p* = 0.041) in the ΔBMI < median group than in the ΔBMI ≥ median group. Other than LDL-C and AT–LDL levels, no significant differences were found among the ΔBMI groups regarding the changes in values before and after cessation.

**Table 2:** Patient data before smoking cessation: Comparison between patients with smaller versus larger BMI changes

	ΔBMI (%) < median^a^	ΔBMI (%) ≥ median
Male/female	71/20	63/29
Age (years)	62 ± 11	60 ± 12
Daily cigarette consumption (*n*)	24 ± 13	24 ± 11
Smoking years	40 ± 11	38 ± 11
FTND score	6.7 ± 1.8	7.0 ± 1.9
Medications		
Antihypertensive agents (%)	17	21
Statins (%)	28	37
Medications for diabetes mellitus (%)	16	12

Data are presented as the mean ± standard deviation

*FTND* Fagerström test for nicotine dependence

^a^Median ΔBMI = 1.25 %

**Table 3:** Patient data before and after successful smoking cessation: comparison between patients with smaller versus larger BMI changes

	ΔBMI (%) < median^a^		ΔBMI (%) ≥ median		*p* value^b^ Time × group
	Baseline	3 months	Baseline	3 months	
BMI (kg/m^2^)	23.4 ± 3.9	23.1 ± 3.8**	23.3 ± 3.4	24.5 ± 3.6**^†^	<0.001
SBP (mmHg)	129 ± 20	126 ± 19*	132 ± 18	129 ± 18	0.824
DBP (mmHg)	74 ± 11	72 ± 12*	76 ± 10	75 ± 10^†^	0.289
HbA_1c_ (%)	5.9 ± 1.1	5.8 ± 1.0	5.6 ± 0.9	5.7 ± 1.1	0.161
LDL-C (mg/dL)	111 ± 30	110 ± 32	115 ± 27	124 ± 27**^‡^	0.009
HDL-C (mg/dL)	56 ± 17	59 ± 17*	57 ± 17	61 ± 19**	0.312
TG (mg/dL)	157 ± 80	189 ± 108*	180 ± 116	217 ± 146**	0.766
SAA-LDL (μg/mL)	20.5 ± 27.4	18.1 ± 18.9	17.6 ± 14.5	25.4 ± 49.8	0.087
AT–LDL (μg/mL)	2.8 ± 1.3	2.3 ± 0.8**	2.6 ± 1.3	2.4 ± 0.9	0.041
CO (ppm)	17.8 ± 18.6	1.6 ± 1.4**	17.4 ± 9.5	2.0 ± 1.5**	0.727
SDS test score	38.8 ± 11.6	37.3 ± 11.3	38.0 ± 10.2	37.7 ± 10.6	0.378

Data are presented as the mean ± standard deviation

*BMI* body mass index, *SBP* systolic blood pressures, *DBP* diastolic blood pressures, *HbA*_*1c*_ hemoglobin A_1c_, *LDL-C* low-density lipoprotein cholesterol, *HDL-C* high-density lipoprotein cholesterol, *TG* triglycerides, *SAA-LDL* serum amyloid A-LDL, *AT–LDL* α1-antitrypsin–low-density lipoprotein, *CO* carbon monoxide, *SDS* test score, self-rating depression scale test score

* *p* < 0.05, ** *p* < 0.01 vs. baseline

^†^ *p* < 0.05, ^‡^ *p* < 0.01 vs. ΔBMI (%) < median

^a^Median ΔBMI = 1.25 %

^b^Two-way analysis of variance to determine the interaction between time [baseline and 12-week] and group [ΔBMI (%) < median and ΔBMI (%) ≥ median]

## Discussion

Quitting smoking has many benefits, one of which is a decline in the risk of a cardiovascular events such as stroke or heart attack [22]. However, weight gain after smoking cessation is a common challenge that can counteract the benefits of smoking cessation [2]. One study found that when persons without diabetes ceased smoking for >4 years, even if they gained weight, there was still a significant reduction in cardiovascular risk compared with before quitting; in addition, it was found that the longer the smoking cessation period, the greater the reduction in cardiovascular events [5]. On the other hand, for persons with diabetes who quit smoking for >4 years, there was a significant decrease in the cardiovascular risk compared with current smokers only if they held their weight gain to <5 kg [5]. The detailed relationship between early-period weight gain after smoking cessation and cardiovascular risk is clearly complex and not fully understood.

Previous large-scale studies have already confirmed the increase in LDL-C levels as a cardiovascular risk marker [23, 24]. In the present study, post-smoking cessation LDL-C levels significantly increased as a whole (*p* < 0.05). As no changes were recognized for the subgroup with the smaller BMI increase, we believe that LDL-C levels were strongly affected by obesity (i.e., weight gain). On the other hand, reduction in HDL-C levels is related to an increase in cardiovascular risk and is associated with smoking and obesity. In our present study, HDL-C levels increased after smoking cessation in the group with the larger BMI increase as well as in the group with the smaller BMI increase. These findings suggest that the post-smoking cessation increase in HDL-C levels is evidence that the favorable impact of quitting smoking outweighs the negative impact of weight gain.

Oxidative stress from smoking is accompanied by an increase in various inflammatory markers that are linked with an increased cardiovascular risk [25]; one such marker is high-sensitivity C-reactive protein levels [26–29]. Although it takes 5 years after smoking cessation to achieve a significant decrease in these inflammatory markers, it has been reported that these markers decrease to nonsmoker levels 20 years after cessation [30]. Similarly, we have previously reported that serum AT–LDL levels are increased in current smokers compared with former and nonsmokers, but there is a rapid decline within the first 3 months after quitting smoking [14]. The present study is consistent with this report. These findings suggest that serum AT–LDL value is a cardiovascular marker that sensitively reflects the smoking-induced oxidative state.

In the group of patients who experienced the smallest degree of weight gain or even a weight loss after smoking cessation, LDL-C levels did not change. Therefore, the decrease in the AT–LDL level seems to be the only marker to accurately reflect the reduction of oxidative stress by smoking cessation. As for the group who experienced a greater degree of weight gain, these subjects showed no change after smoking cessation in AT–LDL levels. One possible explanation for this finding is that because there is an increase in oxidative stress because of weight gain [31], the weight gain may attenuate the decrease in oxidatively modified AT–LDL resulting from smoking cessation. Another possibility is that the increase in LDL-C levels in this group contributed to the failure of the AT–LDL levels to decrease. Either way, for patients who show early-period weight gain after smoking cessation, it is possible that stricter weight control may lead to a decrease in serum AT–LDL, and that, in turn, could decrease cardiovascular risk.

The findings of the present study suggest that a decrease in serum AT–LDL after quitting smoking is influenced by a BMI increase after smoking cessation. Our research results may be useful for promoting high-quality smoking cessation treatment. However, there are several limitations to this study. First, the focus of the present research was AT–LDL, a modified LDL that promotes atherosclerosis; however, no investigation was made of the endpoints of cardiovascular diseases per se. Second, we employed blood samples obtained 2–3 h after a meal. Serum levels of most markers of lipids and adipocytokines can be affected by a meal; however, in our previous report employing similar blood samples, the AT–LDL level sensitively reflected smoking states. Third, our subject pool numbered only 183. In the future, it will be necessary to increase the number of participants and to conduct long-term observations on cardiovascular events.
